# Geographic range velocity and its association with phylogeny and life history traits in North American woody plants

**DOI:** 10.1002/ece3.3880

**Published:** 2018-02-05

**Authors:** Paul G. Harnik, Hafiz Maherali, Joshua H. Miller, Paul S. Manos

**Affiliations:** ^1^ Department of Earth and Environment Franklin and Marshall College Lancaster PA USA; ^2^ Department of Integrative Biology University of Guelph Guelph ON Canada; ^3^ Department of Geology University of Cincinnati Cincinnati OH USA; ^4^ Department of Paleobiology National Museum of Natural History Smithsonian Institution Washington DC USA; ^5^ Department of Biology Duke University Durham NC USA

**Keywords:** biotic velocity, geographic range, macroecology, macroevolution, paleoecology, phylogenetic comparative methods, pollen, Quaternary, Reid's paradox

## Abstract

The geographic ranges of taxa change in response to environmental conditions. Yet whether rates of range movement (biotic velocities) are phylogenetically conserved is not well known. Phylogenetic conservatism of biotic velocities could reflect similarities among related lineages in climatic tolerances and dispersal‐associated traits. We assess whether late Quaternary biotic velocities were phylogenetically conserved and whether they correlate with climatic tolerances and dispersal‐associated traits. We used phylogenetic regression and nonparametric correlation to evaluate associations between biotic velocities, dispersal‐associated traits, and climatic tolerances for 28 woody plant genera and subgenera in North America. The velocities with which woody plant taxa shifted their core geographic range limits were positively correlated from time step to time step between 16 and 7 ka. The strength of this correlation weakened after 7 ka as the pace of climate change slowed. Dispersal‐associated traits and climatic tolerances were not associated with biotic velocities. Although the biotic velocities of some genera were consistently fast and others consistently slow, biotic velocities were not phylogenetically conserved. The rapid late Quaternary range shifts of plants lacking traits that facilitate frequent long‐distance dispersal has long been noted (i.e., Reid's Paradox). Our results are consistent with this paradox and show that it remains robust when phylogenetic information is taken into account. The lack of association between biotic velocities, dispersal‐associated traits, and climatic tolerances may reflect several, nonmutually exclusive processes, including rare long‐distance dispersal, biotic interactions, and cryptic refugia. Because late Quaternary biotic velocities were decoupled from dispersal‐associated traits, trait data for genera and subgenera cannot be used to predict longer‐term (millennial‐scale) floristic responses to climate change.

## 1 INTRODUCTION

The geographic ranges of species can change rapidly. This is evident in the recent shifts of species upslope along elevational gradients (Chen, Hill, Ohlemüller, Roy, & Thomas, 2011; Colwell, Brehm, Cardelús, Gilman, & Longino, 2008), poleward along latitudinal gradients (Breed, Stichter, & Crone, 2013), and in the geographic spread of invasive species (Sakai et al., 2001; Sax, Stachowicz, & Gaines, 2005) over decadal to centennial time scales. Geographic ranges are also dynamic over geologic time, as evidenced by shifts in range locations in response to long‐term climatic changes (Blois & Hadly, 2009; Jackson & Overpeck, 2000; Ordonez & Williams, 2013; Roy, Jablonski, & Valentine, 1995) and the waxing and waning of range sizes over the evolutionary histories of lineages (Foote et al., 2007; Liow & Stenseth, 2007).

Although realized geographic ranges of species can shift rapidly, the rates of these movements (hereafter referred to as “biotic velocities” following Ordonez & Williams, 2013) could still be phylogenetically structured. Such structuring would have important implications for predicting geographic range dynamics in response to future environmental change. For example, lineages with consistently slow biotic velocities may be at a higher risk of extinction, assuming that they also have narrower climatic tolerances. This information could also be used to improve model forecasts for community composition in response to climate change. Addressing whether biotic velocities are phylogenetically structured is challenging because of limited and/or inconsistently measured data on range shifts for current populations and taxa (Lustenhouwer, Moran, & Levine, 2017). Furthermore, biogeographic processes such as rare, long‐distance dispersal are unlikely to be observed with the data available for current populations and taxa.

Nevertheless, indirect evidence from analyses of geographic range size suggests that phylogenetic structuring of biotic velocity could be widespread. Closely‐related lineages could exhibit more similar range sizes than expected by chance because of shared ancestry (as discussed in Vamosi & Vamosi, 2012), but it is also possible that the range sizes of closely‐related lineages differ more than expected because speciation causes sister species to have different initial range sizes. Phylogenetic conservatism in range size has been observed in studies that have compared sister taxa (Jones, Sechrest, & Gittleman, 2005) and ancestor‐descendant pairs (Hunt, Roy, & Jablonski, 2005; Jablonski, 1987), as well as in broad‐scale comparative analyses (Harnik, Fitzgerald, Payne, & Carlson, 2014; Hopkins, 2011; Morin & Lechowicz, 2013; Zacaï et al., 2017). Phylogenetic conservatism in range size has also been observed in a diversity of clades at different times in Earth's history (e.g., Cambrian trilobites (Hopkins, 2011), Devonian brachiopods (Harnik et al., 2014), Jurassic and Cretaceous mollusks (Hunt et al., 2005; Jablonski, 1987; Zacaï et al., 2017), and present‐day mammals (Jones et al., 2005) and trees (Martin & Husband, 2009; Morin & Lechowicz, 2013), at different taxonomic levels (i.e., species [e.g., Hopkins, 2011] and genera [e.g., Harnik et al., 2014]), and in both the marine and terrestrial realms.

Phylogenetic conservatism of life history traits and climatic tolerances could produce phylogenetic conservatism of biotic velocities. Specifically, traits that are associated with growth rate, generation time, and the likelihood of dispersal can be phylogenetically conserved (Chave et al., 2009; Moles et al., 2005; Swenson & Enquist, 2007) and may influence range movement (Stahl, Reu, & Wirth, 2014). For instance, plants with lighter seeds, shorter generation times, and faster growth could, all else being equal, have faster rates of range expansion. Similarly, plants tolerant of cooler temperatures, and those with greater overall environmental breadth, could exhibit faster Quaternary velocities following deglaciation. If these traits are phylogenetically conserved, they could result in similarities in biotic velocities over macroevolutionary time scales (e.g., Böehning‐Gaese, Caprano, van Ewijk, & Veith, 2006), such that closely‐related taxa with shared traits that enable faster and farther dispersal will move more quickly than taxa with more limited dispersal capabilities and/or environmental tolerances. Biotic velocities could be phylogenetically conserved even if the specific individual traits examined in a given study are not, simply because other heritable but unmeasured characteristics influence range movement.

Here we use the geographic range dynamics of woody plants in North America following the last glacial maximum to assess the extent that climatically driven geographic responses of seed plants were affected by shared evolutionary history, life history traits, and environmental tolerances. Quaternary (2.59–0 Ma) fossil records have been widely used to understand the movements of plants (Jackson & Overpeck, 2000; Ordonez & Williams, 2013; Williams, Shuman, Webb, Bartlein, & Leduc, 2004) and animals (Blois & Hadly, 2009; Graham et al., 1996; Roy et al., 1995) in response to climate change because such records have excellent preservation, high temporal and spatial resolution (relative to older geologic intervals), and high taxonomic overlap with modern floras. Previous studies have analyzed Quaternary ranges to determine whether species responded individualistically to changing environmental conditions (Jackson & Overpeck, 2000; Lyons, 2003; Roy et al., 1995; Williams et al., 2004) and whether the pace at which species shifted their ranges was set by late Quaternary climate change (Ordonez & Williams, 2013) and affected by species interactions (e.g., mycorrhizal associations [Lankau, Zhu, & Ordonez, 2015]). Insights gleaned from past range dynamics may be useful in predicting the effects of current and future environmental changes on the diversity and composition of terrestrial ecosystems (Veloz et al., 2012; Williams & Jackson, 2007; Williams, Post, Cwynar, Lotter, & Levesque, 2002), and avoid some of the challenges inherent in studies of range movement in contemporary populations; specifically, having to assume that velocities measured over short time scales hold over longer time scales, and that dispersal‐related traits reliably predict potential velocities when empirical velocity data are unavailable (Lustenhouwer et al., 2017).

Few studies of late Quaternary pollen records have considered the range movements of plants in an evolutionary context (although see Lankau et al., 2015). In most analyses, the geographic dynamics of plants through the late Quaternary are assumed to be phylogenetically independent. However, if dispersal‐related traits are phylogenetically conserved, we might expect range movements to also exhibit phylogenetic signal, although such signal may be somewhat muted as range shifts reflect the contributions of species interactions and other extrinsic biotic and abiotic conditions along the range margin as well as plant dispersal traits and climatic tolerances. Here we use late Quaternary pollen records to address three questions: (1) Do lineages exhibit consistent differences in biotic velocities through time? (2) Do more closely‐related lineages exhibit similar (i.e., phylogenetically conserved) long‐term biotic velocities? (3) Can variation among lineages in late Quaternary biotic velocities be explained by life history traits and climatic tolerances that are in some cases associated with present‐day geographic range limits?

We show that although the North American woody taxa we sampled had similar range dynamics over millennia, there is no significant phylogenetic signal in either biotic velocities or present‐day range sizes. In addition, we found that traits which are correlated with the pace of geographic range shifts today (e.g., seed mass in Hamilton et al., 2005) and present‐day range limits (e.g., cold tolerance in Lee‐Yaw et al., 2016) were not associated with biotic velocities through the late Quaternary. This lack of association may reflect the nonmutually exclusive influences of rare long‐distance dispersal events, biotic interactions, and/or cryptic refugia on late Quaternary biotic velocities.

## 2 MATERIALS AND METHODS

**Figure 1:**
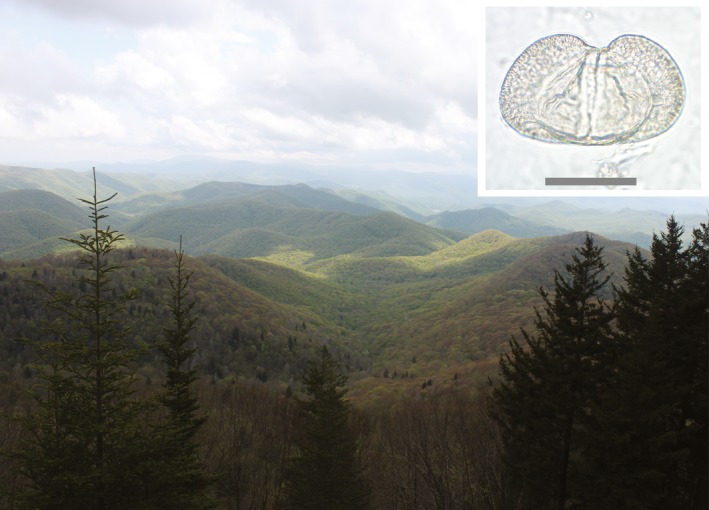
Contemporary vegetation in western North Carolina, with red spruce (*Picea*) and American Beech (*Fagus*) growing in the foreground, and a canopy of oaks (*Quercus*), maples (*Acer*), and other plants in the background. Palynological records (Inset: *Picea* pollen grain, scale bar = 50 μm) reveal dynamic changes in vegetation across North America through the late Quaternary. We assess whether the pace at which woody plants shifted their geographic ranges through the late Quaternary were phylogenetically conserved and whether climatic tolerances and life history traits associated with dispersal were correlated with the rates of these range shifts (biotic velocities). Landscape photograph was taken by Paul Manos at Wolf Mountain Overlook on the Blue Ridge Parkway east of Silva, North Carolina. Inset photograph of *Picea* pollen taken by Mario Williams

We analyzed the late Quaternary biotic velocities of 28 North American woody plant genera and subgenera using velocities estimated by Ordonez and Williams (2013) from fossil pollen records (Figure 1). Fossil pollen have figured prominently in studies of Quaternary range dynamics because of their spatiotemporal resolution and ecological fidelity, despite somewhat coarse taxonomic resolution; many palynological studies focus on the range dynamics of genera rather than species because of the difficulties in distinguishing some species of plants based solely on pollen morphology (e.g., Davis & Shaw, 2001; Delcourt & Delcourt, 1987). Comparative phylogenetic analyses of genus‐level data preclude inquiry into the eco‐evolutionary dynamics of species, yet the benefits of working with empirical estimates of biotic velocities over broad spatiotemporal scales outweigh this limitation. Furthermore, understanding whether genus‐level biotic velocities are phylogenetically conserved is relevant for interpreting the extensive Quaternary paleoecology literature on plant range shifts. While the study of a single, completely‐sampled clade would be optimal, our sample contains two subclades with near complete sampling of the genus‐level diversity relevant to North America: Pinaceae (*Abies*,* Picea*,* Pinus*, and *Tsuga*) and Fagales (*Alnus*,* Betula*,* Castanea*,* Carya*,* Corylus*,* Fagus*,* Juglans*,* Ostrya‐Carpinus*, and *Quercus*).

Ordonez and Williams (2013) calculated the rate at which woody plant genera and subgenera (hereafter referred to as genera following Ordonez and Williams (2013)) shifted the northern and southern boundaries of their core ranges (km/decade) during six time intervals: 16–14 thousand years ago (ka), 14–12 ka, 12–10 ka, 10–7 ka, 7–4 ka, 4–1 ka. These six intervals are approximately equal in duration, although increase slightly in duration towards the present. Biotic velocities were calculated only for genera known from 10 or more locations in a given time interval. Ordonez and Williams focused on the latitudinal range bracketed by the 5th and 95th quantiles of each taxon's occurrences to ensure that these biotic velocities reflected shifts in the core ranges of genera and were not distorted by geographic outliers. Delimiting the core ranges of plant taxa is important as pollen, particularly that of wind‐dispersed plants, which comprise 21 of the 28 genera analyzed here, commonly occurs over much greater geographic areas than standing populations (Bradshaw & Webb, 1985), and thus pollen occurrence in a given core sample is not definitive evidence of a taxon's local presence. Growing evidence for cryptic refugia (e.g., Birks & Willis, 2008; Gavin et al., 2014) provides additional impetus for analyzing core range movements rather than the less well constrained geographic extremes of taxa. As such, shifts in core range are conservative estimates of range change. As with other geographic variables such as range size, realized biotic velocities reflect the interacting contributions of taxon climatic tolerances and dispersal‐related traits—which may be phylogenetically conserved—as well as the suitability of biotic and abiotic environments that populations encounter along the range margin. In keeping with previous palynological analyses, biotic velocities were calculated for genera as species‐level identifications are often not possible.

Because biotic velocities may also be correlated with range size, we tested whether present‐day range sizes are phylogenetically conserved and whether there are associations between present‐day range sizes and late Quaternary velocities. Present‐day geographic ranges of genera in North America were estimated using ESRI ArcGIS 10.0 by overlaying and merging digitized species‐level geographic range maps derived from the Atlas of United States Trees (http://esp.cr.usgs.gov/data/little, accessed August 2013). Range maps for the two North American *Sarcobatus* species were digitized from range maps in the Flora of North America (http://www.efloras.org/, accessed August 2013). Shapefiles of geographic ranges were transformed to an equal area projection (Goode homolosine) before calculating geographic range size and were logarithmically transformed prior to analysis.

To determine whether life history traits associated with contemporary rates of range movement also correlate with millennial‐scale biotic velocities through the late Quaternary, we compiled data for average seed mass, maximum stem diameter, maximum longevity, minimum juvenile period, and wood density. These traits were selected to target a variety of life history strategies that can affect dispersal, from short‐lived plants that produce many, light seeds and maximize rapid reproduction over growth, to plants that maximize long‐term growth but produce fewer, heavier seeds in a given reproductive cycle. For each genus, we calculated the mean trait value for their constituent species in North America (see Data S1). Trait data were unavailable for some species, thus not all constituent species within particular genera are included. Wood density data were obtained from the global wood density database (Chave et al., 2009; Zanne et al., 2009), and seed mass, stem diameter, longevity, and juvenile period data were obtained from Moles, Falster, Leishman, and Westoby (2004), which is derived from the Kew Seed Information database and other sources (see Moles et al., 2004 for further details). Ideally, trait values would be generated from the same time intervals over which biotic velocities were estimated, however, that is not possible because many of these traits are not preserved in the fossil record. Consequently, as in many previous macroecological and macroevolutionary studies, life history traits and climatic tolerances were estimated using contemporary data and assumed to have not changed markedly through the late Quaternary.

Nineteen bioclimatic variables (Hijmans, Cameron, Parra, Jones, & Jarvis, 2005; 10 min rasters) describing present‐day temperature and precipitation patterns were used to establish the range of environmental conditions experienced by each genus over its current geographic range in North America. To determine whether climatic tolerances associated with present‐day range limits (Lee‐Yaw et al., 2016) were associated with late Quaternary biotic velocities, we calculated the median, minimum, and range of mean annual temperatures that each genus experiences today over its geographic range. The minimum temperature tolerance of a taxon may be a particularly important determinant of higher latitude range limits (Kollas, Körner, & Randin, 2014) and thus more likely to show correspondence with northern boundary biotic velocities following the Last Glacial Maximum. In addition, we estimated the overall environmental tolerance of each genus using the multivariate dispersion of all 19 bioclimatic variables, which include both temperature and precipitation patterns. Multivariate dispersion was calculated after mean‐centering and standardizing data for each bioclimatic variable across the combined geographic coverage of all genera. Dispersion was calculated using the *betadisper* function in the vegan package in R (Oksanen et al., 2016; R Development Core Team, 2016). We hypothesized that plants tolerant of cooler temperatures, and those with greater overall environmental breadth, would exhibit faster Quaternary velocities following deglaciation, provided they also had sufficient dispersal ability.

For comparative analyses, an ultrametric phylogenetic tree for all genera with late Quaternary velocity estimates was derived from the APG III seed plant phylogeny reference tree (version 12; Angiosperm Phylogeny Group, 2009) that is contained within the tree building software package, Phylomatic (http://phylodiversity.net/phylomatic; Webb, Ackerly, & Kembel, 2008). The APG supertree has been widely used in plant comparative phylogenetic analyses (e.g., Lustenhouwer et al., 2017). Two pairs of closely‐related plants with pollen morphologies that cannot be consistently distinguished (Ordonez & Williams, 2013) were each collapsed to a single tip in all of our comparative analyses: *Juniperus* and *Thuja*, and *Ostrya* and *Carpinus*, respectively. In the trimmed APG III reference tree (Figure S1), *Juniperus* and *Thuja* are sister taxa, and *Ostrya* and *Carpinus* occur in a polytomy with *Alnus* and *Betula*. The APG III reference tree was temporally‐calibrated using fossil calibrations from Wikström, Savolainen, and Chase (2001) (Figure S1). For our calculations of phylogenetic signal, polytomies were randomly resolved using the *multi2di* function in the *ape* package in R (Paradis, Claude, & Strimmer, 2004), and Pagel's λ (Freckleton, Harvey, & Pagel, 2002; Pagel, 1999) was estimated for all biotic velocities, life history traits, and climatic tolerances using the *fitContinuous* function in the *geiger* package (Harmon, Weir, Brock, Glor, & Challenger, 2008); this process was repeated 100 times and the mean λ values recorded for each trait. Note that resolving polytomies in this way does not affect branch lengths and consequently maximum likelihood estimates of Pagel's λ do not vary. We used Pagel's λ to quantify phylogenetic signal, because it has been shown to be robust to branch length uncertainty, and specifically many of the calibration issues that affect supertrees, including the APG tree used here (Molina‐Venegas & Rodríguez, 2017; Münkemüller et al., 2012). The *phylosignal* R package (Keck, Rimet, Bouchez, & Franc, 2016) was used to plot each of the figures that present mean velocities along the tips of the trimmed phylogeny.

To assess the relative conservatism of biotic velocities within North American lineages we assessed the temporal autocorrelation in biotic velocities, by calculating the Spearman rank order correlation of velocities in one interval and velocities in the subsequent time interval, for each of the six intervals identified above. This approach for assessing conservatism in geographic range characteristics has been used in previous studies (Hadly, Spaeth, & Li, 2009). We used phylogenetic generalized least squares (pgls) regression (Grafen, 1989; Hansen & Bartoszek, 2012) to assess the associations between life history traits, climatic tolerances, and biotic velocities while accounting for shared evolutionary history, and Pagel's λ (Freckleton et al., 2002; Pagel, 1999) to summarize the phylogenetic signal in late Quaternary velocities and present‐day range sizes among North American representatives of these taxa. Because species richness within genera could also influence the biotic velocities, we included the current North American species richness of each genus as a covariate in each phylogenetic regression model. Species richness estimates were derived from Atlas of United States Trees (http://esp.cr.usgs.gov/data/little, accessed August 2013) and Flora of North America (http://www.efloras.org/, accessed August 2013). For the phylogenetic comparative analyses, we calculated arithmetic mean velocities for each taxon across a predefined temporal interval, that is, (1) all six intervals (16–1 ka), (2) intervals >7 ka which were characterized by relatively fast velocities, and (3) intervals <7 ka characterized by relatively slow velocities (Figure 1). Because our results were similar for each of the three velocity estimates, we present the results for mean biotic velocities for intervals >7 ka here and include the results for mean velocities for 16–1 ka and <7 ka in the [Link], [Link], [Link], [Link], [Link], [Link]. Phylogenetic comparative analyses were limited to those genera with both velocity and life history trait estimates, and consequently the sample size of genera varies among our comparative analyses. Because of this sample size limitation, we were unable to account for covariation among traits and climatic tolerances using a single multivariate regression model and instead fit separate phylogenetic regression models to the subset of genera for which we had both trait and velocity estimates.

All data analyzed, including the species data used to calculate mean genus traits, are archived in Data S1 and S2).

## 3 RESULTS

**Figure 2:**
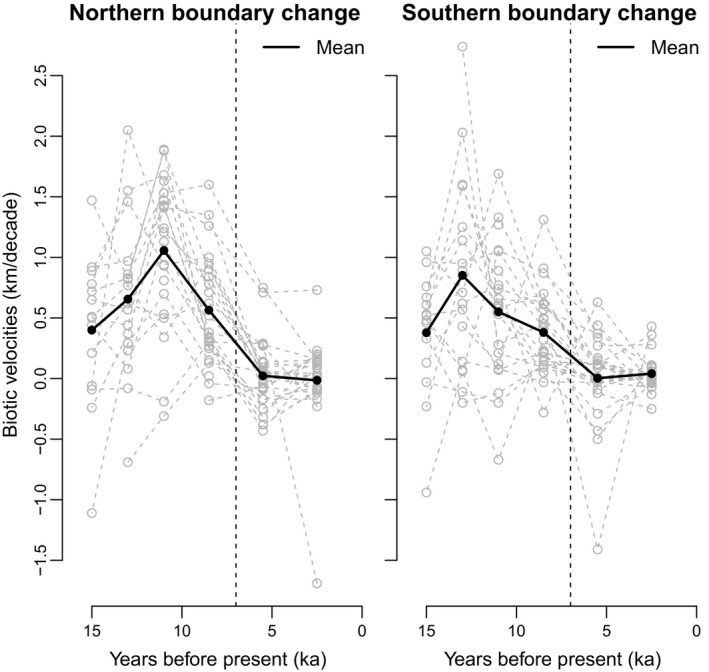
Rates of movement for the northern and southern boundary core range limits of 28 North American woody plant genera over the last 16,000 years. Biotic velocities from Ordonez and Williams (2013). The vertical line separates intervals >7 ka that were characterized by relatively rapid range shifts from intervals <7 ka that were characterized by relatively slow range shifts. The solid black line is the mean rate of range movement over time. Biotic velocities are plotted at the mid‐points of each time interval

**Figure 3:**
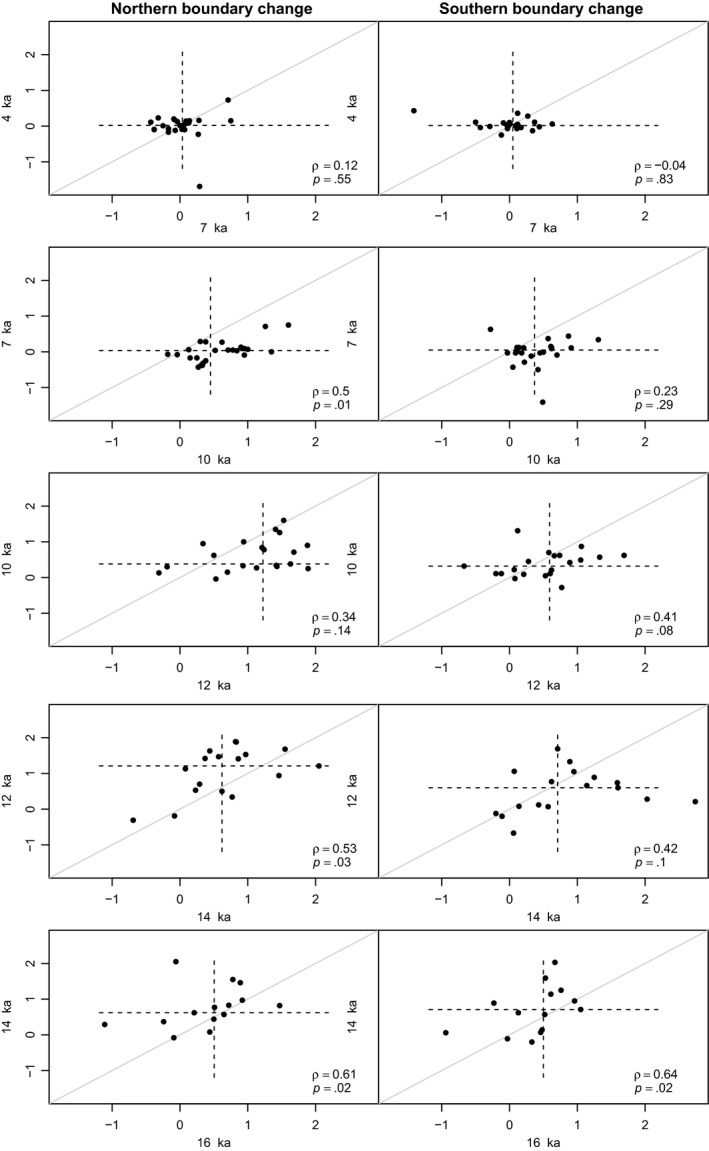
The temporal correlation of woody plant biotic velocities from interval to interval through the late Quaternary. Velocities in a given interval tend to be significantly correlated with velocities in the preceding interval, with the strength of this correlation weakening towards the present‐day as velocities slowed and the variance in velocities declined. Panels plot northern and southern boundary velocities (km/decade) for adjacent time intervals. Points are plant genera with velocity estimates in both intervals. The solid gray line is the 1:1 line. Dashed horizontal and vertical lines are median values. Spearman ρ values and *p* values are noted in the lower‐right corner of each panel. Axes are labeled with the age at the base of each time interval in thousands of years (ka)

**Figure 4:**
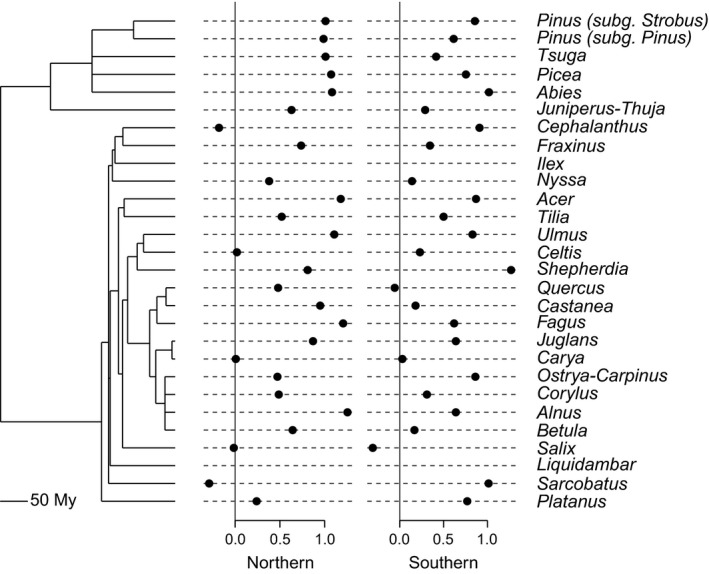
Phylogeny of North American woody plants trimmed to include only those genera with late Quaternary biotic velocities calculated from fossil pollen records. Plotted at the tips are mean “fast interval” (>7 ka) biotic velocities (km/decade) for the northern and southern core range boundaries; *Ilex* and *Liquidambar* biotic velocities were not calculated in “fast intervals” because of the limited geographic occurrences of these two taxa at those times (Ordonez & Williams, 2013). My, millions of years

The biotic velocities of North American woody plants varied over the last 16,000 years (Figure 2). Relatively rapid range shifts occurred following deglaciation with a decline in velocities towards the present‐day. Despite such temporal variation, some plant lineages tended to be consistently rapid movers (e.g., *Acer*,* Alnus*, and *Fagus*) whereas others tended to move more slowly (e.g., *Celtis*,* Nyssa*) (Figures 3 and 4). Biotic velocities in a given 2 kyr time interval tended to be positively correlated with biotic velocities in the subsequent interval (Figure 3). Temporal autocorrelation was generally stronger for the northern versus southern edges of plant core ranges (Figure 3). Temporal autocorrelation weakened over time (Figure 3) as biotic velocities slowed in tandem with climatic velocities (Ordonez & Williams, 2013).

In contrast, Quaternary biotic velocities are phylogenetically independent at the broader scale of North American woody plants (Figures 4, S2, and S3); mean biotic velocities for both the northern and southern range edges are phylogenetically independent (Figure 4, Table 1). These results are unaffected by the time span over which mean velocities were calculated; mean velocities calculated for “fast” intervals (i.e., > 7ka), “slow” intervals (i.e., <7 ka), or all intervals combined are phylogenetically independent (Table 1). It appears, therefore, that over geologic time millennial‐scale biotic velocities change relatively rapidly in extant North American woody plants.

**Table 1:** Maximum likelihood estimate of Pagel's λ for northern and southern edge biotic velocities

	Northern boundary velocity			Southern boundary velocity		
	λ	Weight	*N*	λ	Weight	*N*
Mean for all intervals (16–1 ka)	0.24	0.34	28	0	0.27	26
Mean for “fast” intervals (>7 ka)	0.15	0.29	26	0	0.27	26
Mean for “slow” intervals (<7 ka)	0	0.27	26	0	0.27	26

Results are presented for mean velocities calculated using all intervals, intervals >7 ka, and intervals <7 ka. Weight is the Akaike weight for the model in which λ is estimated relative to a model in which λ = 0. λ values closer to one indicate that velocities are evolving under pure Brownian motion with more closely‐related lineages exhibiting greater trait similarity, and λ values closer to zero indicate that velocities are evolving more independently of the phylogeny (i.e., little phylogenetic signal). Akaike weights summarize the relative support for each model in the set of models under consideration, with values closer to one indicating greater support for that model; lower weights indicate greater support for the model in which λ = 0. *N* is the number of genera with velocity estimates included in that analysis. Pagel's λ values and Akaike weights are mean values calculated from 100 iterations in which polytomies in the trimmed APG phylogeny were randomly resolved. Because of the distribution of these polytomies in the phylogeny, branch lengths were unaffected and consequently there is no variance in Pagel's λ estimates or Akaike weights.

Phylogenetic signal was observed in some genus‐level traits (e.g., maximum longevity, median mean annual temperature), but not others (e.g., average seed mass, maximum stem diameter; Table 2). Yet, phylogenetically generalized regression analysis did not detect significant associations between any plant life history traits and late Quaternary biotic velocities (Table 3). Average seed mass, maximum stem diameter, maximum longevity, minimum juvenile period, and wood density were not associated with either northern or southern edge biotic velocities through the late Quaternary (Figure 4), regardless of whether mean velocities were calculated for intervals (>7 ka) characterized by relatively fast velocities (Table 3), or for intervals (<7 ka) characterized by relatively slow velocities (Table S1). Present‐day climatic tolerances (i.e., multiple measures of temperature tolerance as well as a composite measure of overall environmental breadth) were also not associated with northern or southern edge late Quaternary biotic velocities (Tables 3 and S1). Species richness within genera was also generally not associated with northern or southern edge biotic velocities through the late Quaternary (Table 3), with the exception of two models in which southern edge biotic velocities was the response variable; one model contained median mean annual temperature as a predictor, and the other model contained minimum mean annual temperature (Table 3). In both of these models, there was a weak negative relationship between species richness and biotic velocity after accounting for that aspect of climatic tolerance, but neither of these associations are statistically significant after Bonferroni correction for multiple comparisons. Maximum likelihood estimates of Pagel's λ for almost all trait‐velocity associations were equal to zero, indicating no phylogenetic signal (Table 3).

**Table 2:** Maximum likelihood estimate of Pagel's λ for life history traits and climatic tolerances

	λ	Weight	*N*
Average seed mass	0	0.27	24
Maximum stem diameter	0	0.27	15
Maximum longevity	0.83	0.99	24
Minimum juvenile period	0	0.27	22
Wood density	0.18	0.38	24
Median mean annual temperature	0.47	0.70	28
Minimum mean annual temperature	0.35	0.53	28
Mean annual temperature range	0.14	0.3	28
Multivariate environmental tolerance	0.02	0.28	28

Weight is the Akaike weight for the model in which λ is estimated relative to a model in which λ = 0. λ values closer to one indicate that velocities are evolving under pure Brownian motion with more closely‐related lineages exhibiting greater trait similarity, and λ values closer to zero indicate that velocities are evolving more independently of the phylogeny (i.e., little phylogenetic signal). Akaike weights summarize the relative support for each model in the set of models under consideration, with values closer to one indicating greater support for that model; lower weights indicate greater support for the model in which λ = 0. *N* is the number of genera with trait estimates included in that analysis. Pagel's λ values and Akaike weights are mean values calculated from 100 iterations in which polytomies in the trimmed APG phylogeny were randomly resolved. Because of the distribution of these polytomies in the phylogeny, branch lengths were unaffected and consequently there is no variance in Pagel's λ estimates or Akaike weights.

**Table 3:** Phylogenetic regression results for the associations between life history traits and climatic tolerances and northern and southern edge biotic velocities

Predictor variables	Northern boundary velocity			Southern boundary velocity		
	λ	Cor	*N*	λ	Cor	*N*
Average seed mass	0	0.19	22	0.08	0.17	22
Maximum stem diameter	0.12	0.19	15	0.06	0.45	15
Maximum longevity	0	0.11	22	0	0.41	22
Minimum juvenile period	0.04	0.07	21	0.19	0.11	21
Wood density	0	−0.21	22	0.15	−0.31	22
Median mean annual temperature	0	−0.38	26	0	−0.08*	26
Minimum mean annual temperature	0	−0.24	26	0	0.14*	26
Mean annual temperature range	0.17	−0.02	26	0	−0.22	26
Multivariate environmental tolerance	0.15	0.02	26	0	−0.25	26

Results are presented for mean velocities in “fast intervals” (>7 ka). See Table S1 for results for “slow intervals” (<7 ka). λ is the estimated phylogenetic signal in the residuals for the model. Cor = correlation coefficient calculated from *t* values (Rosenberg, Adams, & Gurevitch, 2000). *N* = number of taxa with velocity and trait data that were included in that model. The *p* values for all estimated coefficients between life history traits and climatic tolerances and northern and southern edge biotic velocities were ≥.05. Note, species richness was used as a covariate in each model to account for the possibility that species richness also influenced genus biotic velocities. *Indicate models in which the estimated partial coefficients between current species richness and northern or southern edge biotic velocities were statistically significant (*p *<* *.05). All estimated partial coefficients between biotic velocities and species richness within genera were not significant, with the exception of the association between southern edge biotic velocities and median mean annual temperature (*p* = .02) and minimum mean annual temperature (*p* = .04). Neither of these associations remains after Bonferroni correction for multiple comparisons.

**Figure 5:**
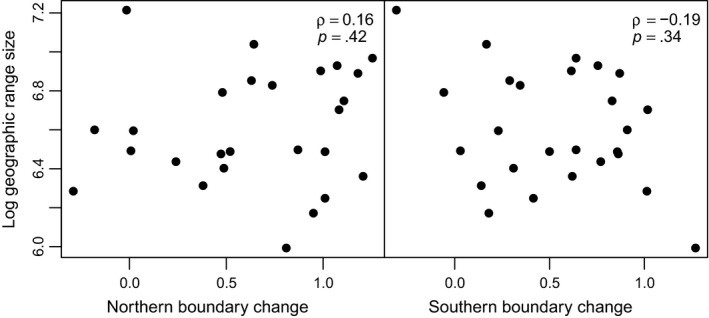
Present‐day geographic range sizes (log geographic area) versus mean “fast interval” (>7 ka) northern and southern edge late Quaternary velocities (km/decade) for North American woody plants. Spearman ρ values and *p* values are noted in the upper‐right corner of each panel

To assess whether the lack of phylogenetic signal in biotic velocities was due to our consideration of rates of geographic movement rather than other geographic range properties, such as overall geographic range size considered by previous studies (e.g., Hunt et al., 2005; Jones et al., 2005), we also estimated Pagel's λ for present‐day range sizes. The maximum likelihood estimate of λ for the present‐day range sizes of all 28 plant genera with late Quaternary velocities equaled zero. In addition, present‐day range sizes were not correlated with mean northern or southern edge velocities through the late Quaternary in North America (Figure 5); for this correlation test, we examined mean velocities for “fast” intervals (>7 ka) for which there was a greater variance in velocities.

## 4 DISCUSSION

We find that the biotic velocities of individual woody plant genera were consistent through the late Quaternary, as evident by their temporal autocorrelation over the past 16 ka. Over time, some plant genera (e.g., *Acer*,* Alnus*, and *Fagus*) tended to be rapid movers whereas others tended to move at a slower pace (e.g., *Celtis*,* Nyssa*). Rank order differences in genus biotic velocities were correlated between adjacent time intervals, although the absolute rates of range movement varied (Figures 2 and 3), and autocorrelation tended to be stronger for northern versus southern velocities. This result is similar to that observed in analyses of North American mammal genera in which late Holocene geographic range sizes were significantly correlated with late Pleistocene range sizes (Hadly et al., 2009). Although the properties (e.g., velocities, size) of genus geographic ranges varied over time (Figure 2), relative differences among genera were maintained for millennia (Figure 3). This temporal autocorrelation may reflect conserved range dynamics within lineages and/or consistent responses of lineages to extrinsic environmental conditions that are shared between adjacent temporal intervals.

Maintenance of biotic velocities over millennia did not extend, however, to the deep time scales recorded by phylogeny. Late Quaternary biotic velocities were independent of phylogeny, with sister taxa in our trimmed phylogeny often exhibiting markedly different mean biotic velocities (Figure 3). This result could reflect the scale‐dependence of geographic dynamics, such that the conservative estimates of late Quaternary velocities analyzed here do not reflect the long‐term range dynamics of these lineages over their evolutionary histories. For example, the mean divergence times of extant seed plant lineages can be two to three orders of magnitude greater than the 16 ka time slice spanned by our velocity estimates (e.g., for extant angiosperms see extended data Figure 2 in Zanne et al. (2014), and for extant conifers see Figure 1 in Leslie et al. (2012)). Alternatively, it is possible that our power to detect phylogenetic signal is limited by the restricted sample size of genera for which we have robust late Quaternary velocity estimates. In select instances, sparse taxon sampling placed genera in different orders (e.g., *Fraxinus* and *Cephalanthus*) on adjacent branches in the trimmed phylogeny (Figure 4). However, the trimmed phylogeny also includes two subclades with near complete sampling of North American genus‐level diversity: Pinaceae (*Abies*,* Picea*,* Pinus*, and *Tsuga*) and Fagales (*Alnus*,* Betula*,* Castanea*,* Carya*,* Corylus*,* Fagus*,* Juglans*,* Ostrya‐Carpinus*, and *Quercus*). More broadly, because the range dynamics of higher taxa are a function of processes of speciation and extinction as well as the physiological tolerances of constituent species (Miller 1997; Roy, Hunt, Jablonski, Krug, & Valentine, 2009), cladogenetic, and anagenetic changes in taxon geographic ranges could lead to the disassociation of biotic velocities and phylogeny over geologic time scales.

Present‐day range limits can be shaped by functional traits as well as climatic tolerances. Hamilton et al. (2005), for example, found that seed mass was inversely correlated with invasion success at regional to continental scales in Australia. In an analysis of European deciduous trees, Randin et al. (2013) found that species latitudinal range limits reflected their thermal tolerances. Our results indicate that many of the life history traits and climatic tolerances that affect the dispersal, demography, and geographic movements of seed plants today are decoupled from realized rates of range movement over past millennia. Although some genera that moved rapidly through the late Quaternary possess traits that are expected to confer rapid biotic velocities (e.g., light seeds, short‐lived, brief juvenile period, and low‐density wood), other genera with quickly changing late Quaternary ranges do not share these adaptations. The relatively rapid late Quaternary velocities of *Acer* and *Alnus*, for example, do not result from similar traits. *Acer* has relatively heavy seeds and a long juvenile period and thus is expected to move relatively slowly, whereas *Alnus* has light seeds and a brief juvenile period and is expected to move relatively fast. *Acer*'s relatively rapid late Quaternary velocities may reflect the winged morphology of its seeds, which enhance the probability of long‐distance dispersal (Nathan et al., 2002). Similarly, although some plants that moved slowly through the late Quaternary, such as *Carya* and *Nyssa,* have heavy seeds and dense wood, which could result in dispersal limitation, the traits of slow movers (e.g., longevity and duration of juvenile period) vary considerably and are often comparable to those of plants that moved at faster rates. The decoupling of life history traits from late Quaternary biotic velocities, and specifically the rapid late Quaternary velocities of plants that possess heavier seeds and other dispersal‐limiting traits has been recognized by others previously as “Reid's paradox” (Birks, 1989; Clark, 1998; Clark et al., 1998; Reid, 1899; Skellam, 1951). Our findings show that “Reid's paradox” has broad empirical support across the North American woody plant flora, even when the effects of shared ancestry are included in the analysis.

There are several non‐mutually exclusive explanations for the decoupling of late Quaternary biotic velocities and life history traits and climatic tolerances. First, apparently rapid rates of postglacial geographic expansion by plants with slower life histories could be due to the occurrence of cryptic refuges proximal to the retreating ice margin (Birks & Willis, 2008; Gavin et al., 2014; Jackson et al., 2000; McLachlan, Clark, & Manos, 2005; although see Tzedakis, Emerson, & Hewitt, 2013). Under this scenario, some genera included in our study (e.g., *Acer* and *Fagus),* may have moved more slowly than pollen records would suggest because low‐density populations remained at higher latitudes. Recent studies combining molecular phylogeographic data with analyses of plant macro‐ and microfossils have identified cryptic refuges (Magri et al., 2006; McLachlan et al., 2005), and suggest that late Quaternary biotic velocities may have been <1 km/decade (McLachlan et al., 2005), considerably less than the 1–10 km/decade reported in previous studies. Note that the biotic velocities calculated by Ordonez and Williams (2013) analyzed here are at the lower end of previously published velocity estimates from fossil pollen, and yet these conservative estimates still show a disassociation between late Quaternary biotic velocities and traits related to dispersal and demography.

The decoupling of life history traits and time‐integrated biotic velocities could also be due to rare long‐distance dispersal events (Clark, 1998). Under this scenario, rare events may have led to the seemingly rapid biotic velocities of plants with generally dispersal‐limiting life history traits. Over millennia, rare long‐distance dispersal events would allow the biotic velocities of plants with slower life histories to catch up geographically with plants with faster life histories, thereby evening out differences in dispersal potential and biotic velocities manifest at finer time scales. Rapid rates of post‐glacial expansion may have also been facilitated by biotic interactions, as has been hypothesized for fagaceous trees and blue jays (Johnson & Webb, 1989), and woody plants and micorhiza type (Lankau et al., 2015). Note that the effects of cryptic refugia, long‐distance dispersal, and biotic interactions are not mutually exclusive, as shown by Giesecke and Bennett (2004) in their study of the post‐glacial expansion of *Picea* in Scandinavia.

It is also possible that life history traits may play less of a role in realized range shifts than is often assumed. Some support for this comes from a recent meta‐analysis of plant and animal studies, which found that although life history traits were associated with present‐day range shifts in some instances, overall trait‐based models had low predictive power (Angert et al., 2011). Life history traits may determine the potential rate of movement for a lineage, yet the realized rate is also affected by biotic interactions, climatic conditions, and other biotic and abiotic environmental parameters along the range edge. This could result in a dissociation between dispersal traits, which can be phylogenetically conserved, and realized biotic velocities. Covariation between life history traits that have opposing effects on dispersal potential could also result in taxa exhibiting similar biotic velocities despite markedly different life histories (Lustenhouwer et al., 2017).

The coarse taxonomic scale (genus‐level) used in our study, which was necessitated by the inability to differentiate pollen to species based on morphology alone, poses some limitations. Congeneric species can vary in range size, location, and rate of geographic range movement and thus the range dynamics of a genus do not scale down to all constituent species (Foote, Ritterbush, & Miller, 2016; Hendricks, Saupe, Myers, Hermsen, & Allmon, 2014; Hoehn, Harnik, & Roth, 2016; Jablonski, 2008). Variation among species in life history, climatic tolerance, and other macroecological traits can lead to genus‐level estimates of these characteristics that differ and are not representative of, those of their congeneric species, resulting in hierarchical differences in the ecological and evolutionary processes of species and higher taxonomic groups (Jablonski, 2007). Moreover, aggregating species data within genera may be sensitive to the completeness of species‐level sampling, which varies among the traits considered here (see Data S2). Previous studies have found that macroecological variables (e.g., range size) can be significantly correlated between morphogenera and molecularly‐defined clades (Jablonski & Finarelli, 2009), which suggests that paraphyletic groups, such as the North American woody plant genera and subgenera analyzed here, may provide robust trait estimates. Furthermore, the geographic range dynamics of higher taxa are a function of physiology, environmental tolerance, and environmental history, but also processes of speciation and extinction. Bridge species—those species that span major environmental barriers—generate some of the broad‐scale variation in the range sizes of higher taxa in the marine and terrestrial realms (Jablonski et al., 2013; Tomašových et al., 2016) and may also affect the biotic velocities of higher taxa over geologic time. Consequently, given the taxonomic resolution of our analyses, we cannot conclude that late Quaternary biotic velocities were decoupled from functional traits at the species‐level. Although it is plausible that processes similar to those discussed above may have been important in the range shifts of species integrated over millennial scales. Future work could address this issue by focusing on a subset of plant lineages in which pollen can be resolved to the species‐level. Comparing the modern geographic distributions of sister taxa within many of the woody North American genera studied here using fossil‐calibrated phylogenies (Manos & Meireles, 2015) could also help to elucidate the heritability of range size across a range of divergence times and environmental tolerances.

We have assumed in this study that the biotic velocities estimated by Ordonez and Williams (2013) reflect biologically meaningful changes in the core ranges of North American woody plants through the late Quaternary. It is conceivable however that issue of pollen preservation and patchy and/or sparse data, among other factors, could lead to inaccurate velocity estimates, which may limit our ability to detect associations with traits of present‐day plants. The impact of uncertainty in biotic velocities cannot be assessed in the present study, but we note that these velocity estimates do covary with other factors, specifically climatic velocities, which are also hypothesized to affect plant range movements (Ordonez & Williams, 2013).

In conclusion, we assessed the relationships between phylogeny, biotic velocities, and life history traits and climatic tolerances, in North American woody plants, a system in which we can integrate data from the late Quaternary fossil record, phylogeny, and functional biology. We found that relative differences among woody plant genera in their biotic velocities were maintained through the Quaternary but were not phylogenetically conserved. Plant functional traits and climatic tolerances were not associated with Quaternary biotic velocities. These results provide a comparative phylogenetic perspective on the geographic range dynamics of woody plants and underscore the temporal scale‐dependence of range shifts for which functional traits may be predictive in the near term but less informative over longer time scales.

## CONFLICT OF INTEREST

None declared.
