# Supplementary figures and images for: Geographic range velocity and its association with phylogeny and life history traits in North American woody plants
Source: Ecol Evol. 2018 Feb 5;8(5):2632–44. doi: 10.1002/ece3.3880 (PMC5838057; doi:10.1002/ece3.3880)

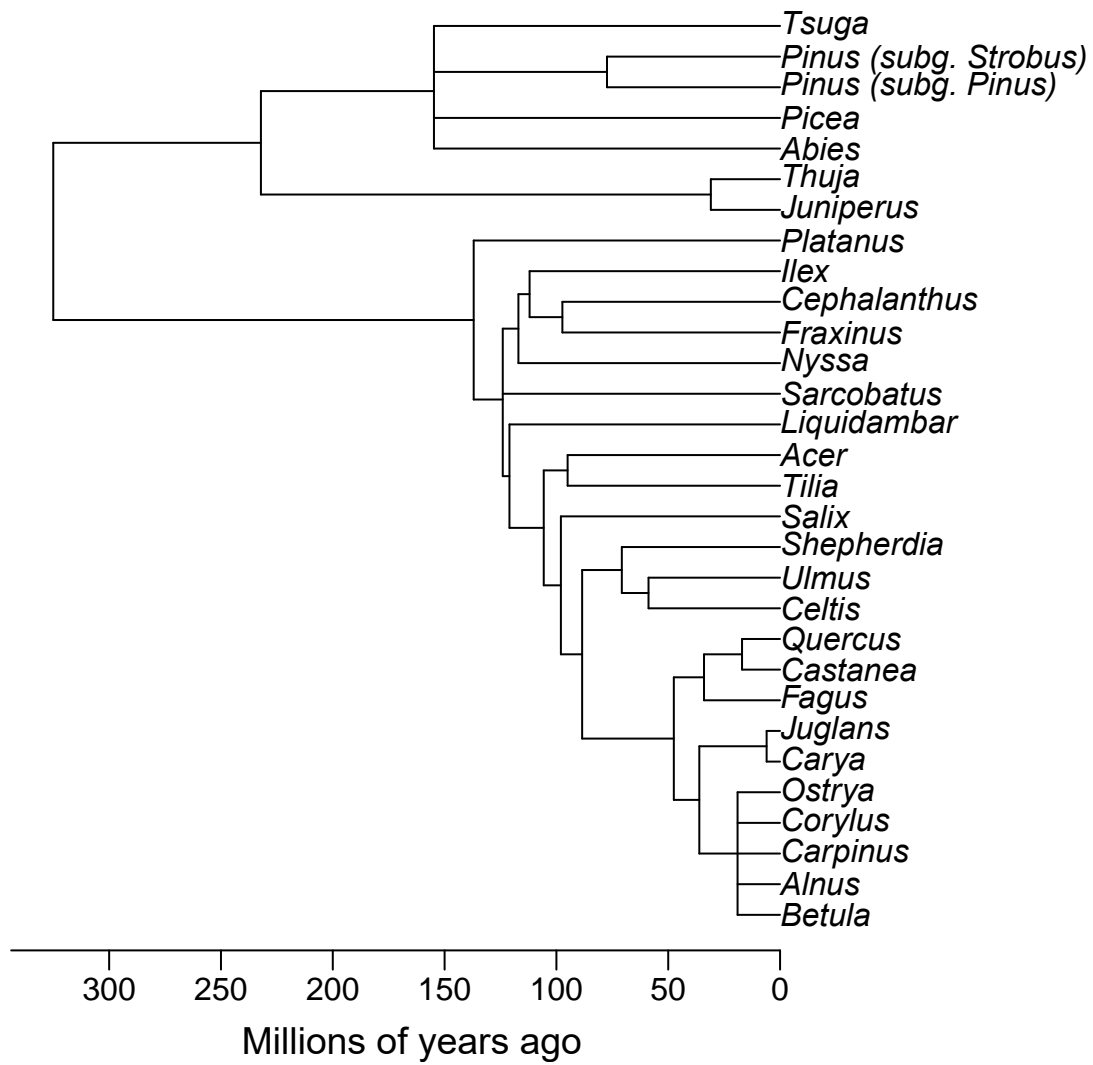

Supplement: Supplementary file 1 [file ECE3-8-2632-s001.pdf]

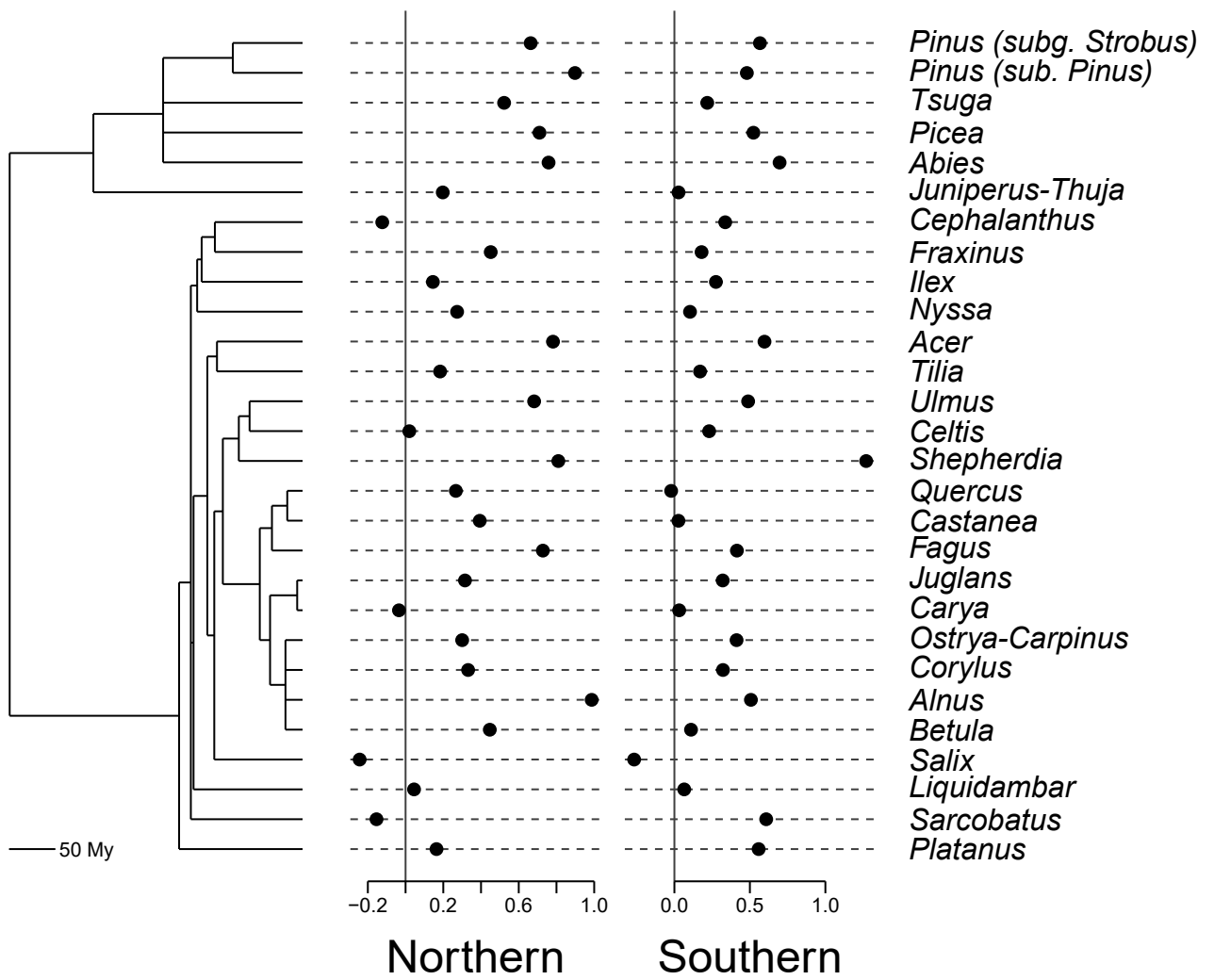

Supplement: Supplementary file 2 [file ECE3-8-2632-s002.pdf]

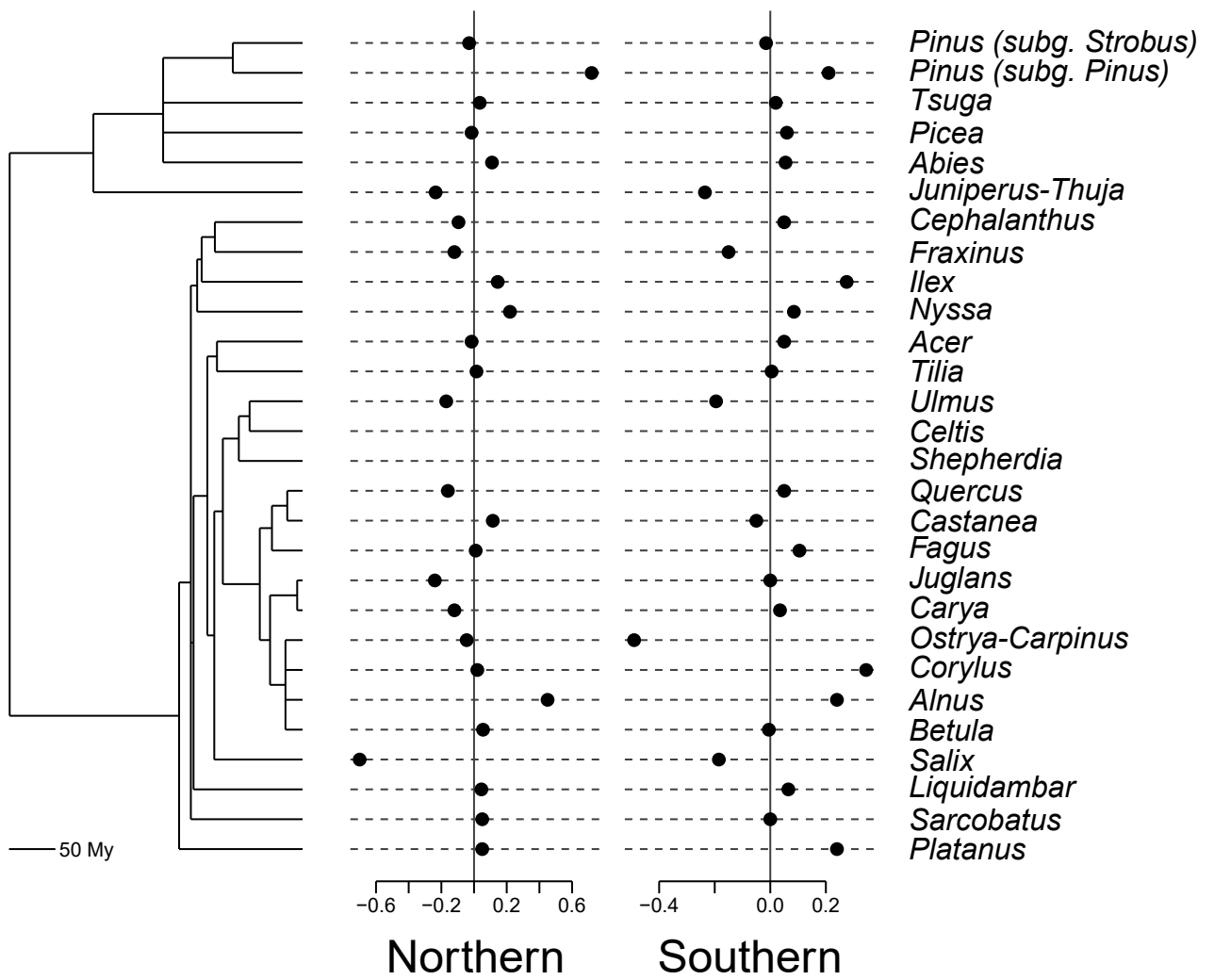

Supplement: Supplementary file 3 [file ECE3-8-2632-s003.pdf]
