# Supplementary material for: Geographic range velocity and its association with phylogeny and life history traits in North American woody plants
Source: Ecol Evol. 2018 Feb 5;8(5):2632–44. doi: 10.1002/ece3.3880 (PMC5838057; doi:10.1002/ece3.3880)
Supplement: Supplementary file 4 [file ECE3-8-2632-s004.docx]

**Table S1.** Phylogenetic regression results for the associations between life history traits and climatic tolerances and northern and southern edge biotic velocities. Results are presented for mean velocities in ‘slow intervals’ (< 7ka). Slope coefficients are unstandardized. SE = one standard error. λ is the estimated phylogenetic signal in the residuals for the model. N = number of taxa with velocity and trait data that were included in that model. The *p* values for all estimated coefficients between life history traits and climatic tolerances and northern and southern edge biotic velocities were ≥ 0.05. Note, current species richness was used as a covariate in all models to account for the possibility that it influenced biotic velocities. None of the estimated partial coefficients between species richness within genera and northern or southern edge biotic velocities were statistically significant.

|  | **Northern Boundary Velocity** | | | **Southern Boundary Velocity** | | |
| --- | --- | --- | --- | --- | --- | --- |
| **Predictor variables** | **Coefficient ± 1 SE** | **λ** | **N** | **Coefficient ± 1 SE** | **λ** | **N** |
| Average seed mass | 0.00 ± 0.00 | 0 | 23 | 0.00 ± 0.00 | 0 | 23 |
| Maximum stem diameter | 0.00 ± 0.00 | 0.06 | 14 | 0.00 ± 0.00 | 0 | 14 |
| Maximum longevity | 0.00 ± 0.00 | 0 | 23 | 0.00 ± 0.00 | 0 | 23 |
| Minimum juvenile period | -0.01 ± 0.01 | 0 | 22 | 0.00 ± 0.00 | 0 | 22 |
| Wood density | -0.34 ± 0.67 | 0 | 23 | -0.39 ± 0.42 | 0 | 23 |
| Median mean annual temperature | -0.31 ± 0.98 | 0 | 26 | 0.09 ± 0.68 | 0 | 26 |
| Minimum mean annual temperature | 0.32 ± 0.77 | 0 | 26 | 0.31 ± 0.53 | 0 | 26 |
| Mean annual temperature range | -0.74 ± 0.74 | 0 | 26 | -0.71 ± 0.50 | 0 | 26 |
| Multivariate environmental tolerance | -0.05 ± 0.10 | 0 | 26 | -0.03 ± 0.07 | 0 | 26 |
